# Electrochemical and DFT insights into 2-amino-4-(4-hydroxy-3-methoxyphenyl)-7-methyl-4*H*-chromene-3-carbonitrile: an innovative strategy for antibacterial activity and corrosion protection of carbon steel

**DOI:** 10.1039/d4ra03785e

**Published:** 2024-08-02

**Authors:** Badreah A. Al Jahdaly

**Affiliations:** a Chemistry Department, Faculty of Science, Umm Al-Qura University 21955 Makkah Saudi Arabia bajahdali@uqu.edu.sa +966504311372

## Abstract

This study explored the potential of a newly synthesized derivative, 2-amino-4-(4-hydroxy-3-methoxyphenyl)-7-methyl-4*H*-chromene-3-carbonitrile (AHMCC), as a broad-spectrum antibacterial agent and a corrosion inhibitor for carbon steel (C.STL) in 0.5 M HCl solution. AHMCC demonstrated remarkable antibacterial efficacy against Gram-negative (*Escherichia coli*, *Klebsiella pneumoniae*) and Gram-positive (*Bacillus subtilis*, *Staphylococcus aureus*) bacteria, as evidenced by agar plate tests and cell viability assays. In the corrosion inhibition studies, AHMCC exhibited mixed-type inhibitor behavior as revealed by potentiodynamic polarization (PDP) measurements. The inhibition efficiency increased with rising AHMCC concentration, confirmed by a significant enhancement in charge transfer resistance (*R*_ct_) observed in electrochemical impedance spectroscopy (EIS) analysis. Electrochemical frequency modulation (EFM) data with obtained CF^2^ and CF^3^ values further corroborated these findings. Langmuir isotherm modeling suggested AHMCC molecules followed a monolayer adsorption pattern on the C.STL surface. UV-visible spectroscopy indicated the formation of a protective layer through chemical interaction between AHMCC and the metal surface. Atomic force microscopy (AFM) provided visual confirmation of this protective film shielding the C.STL from the corrosive environment. Additionally, theoretical calculations supported the proposed adsorption mechanism of AHMCC molecules onto the C.STL surface.

## Abstract 2

This study evaluated AHMCC as a broad-spectrum antibacterial agent and corrosion inhibitor for carbon steel in 0.5 M HCl.

## Graphical abstract

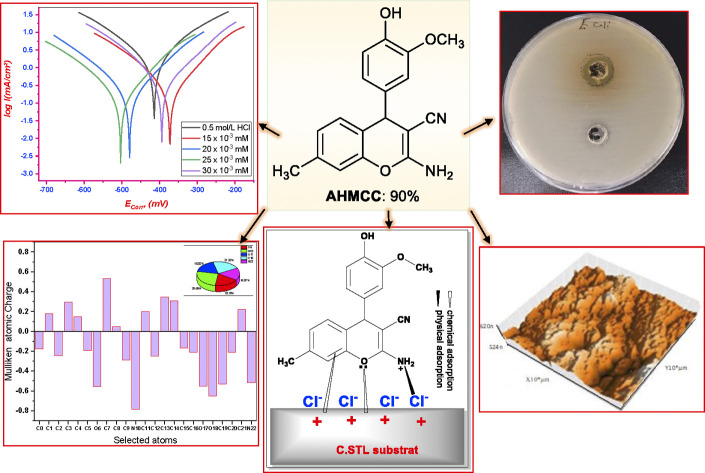

## 1. Introduction

Carbon steel remains a dominant choice for pipelines and containers in the oil and gas industry due to its affordability and exceptional mechanical properties.^1–3^ However, a significant threat lurks – corrosion. This spontaneous degradation of metals in harsh environments poses substantial risks to the integrity, operational efficiency, and financial stability of oil and gas facilities.^4–7^ Highly corrosive environments include acidic solutions like hydrochloric acid (HCl) and sulfuric acid (H_2_SO_4_), as well as salt solutions such as seawater. To combat these detrimental effects and safeguard metal integrity, the oil and gas industry heavily relies on corrosion inhibitors – specialized chemical additives.^4–7^ These inhibitors function by adsorbing onto metal surfaces, forming protective layers. Organic inhibitors, particularly effective in influencing both cathodic and anodic reactions that drive corrosion, are considered a cornerstone of corrosion protection strategies.^8–12^ Organic inhibitor molecules often contain heteroatoms (like oxygen, nitrogen, and sulfur), π-electrons, polar groups, multiple bonds, and aromatic rings. These features serve as reactive centers, facilitating electron donation or acceptance during adsorption onto metal surfaces.^13–17^ The effectiveness of inhibitor adsorption at the metal-solution interface depends on several factors, including the metal's properties, surface charge, the corrosive nature of the electrolyte, and the inhibitor's own chemical structure.^18–20^ Chromenes, recognized for their eco-friendly nature, have found diverse applications across various industries.^21–25^ These include food, dyes, analytical chemistry, catalysis, medicine, fungicides, agrochemicals, and biology. Notably, chromene derivatives incorporating additional heteroatoms (like nitrogen and oxygen) and aryl groups have demonstrated promising potential as corrosion inhibitors for metals exposed to a wide range of aggressive environments.^26–28^ The selection of a 0.5 M HCl solution for this study was guided by its relevance in simulating highly corrosive acidic environments commonly encountered in industrial settings. Hydrochloric acid at this concentration provides a rigorous test for evaluating the performance of corrosion inhibitors, as it is a strong acid that is frequently used in industrial processes and cleaning operations. This concentration is also commonly employed in corrosion studies to ensure consistent and comparable results across different investigations, enabling a comprehensive assessment of the inhibitor's effectiveness. The design of the AHMCC derivative focused on two key objectives: improving its water solubility and enhancing its ability to coordinate with metal surfaces in aqueous environments. Proton nuclear magnetic resonance (^1^H-NMR), Fourier-transform infrared spectroscopy (FT-IR), and melting point measurements were employed to confirm the successful synthesis and chemical structure of the AHMCC inhibitor. This study investigated the dual functionality of the AHMCC derivative: its antibacterial properties and its corrosion inhibition potential. Agar plate tests and cell viability staining techniques were utilized to assess its antibacterial efficacy. To evaluate its effectiveness as a corrosion inhibitor for carbon steel (C.STL) in chloride-containing aqueous solutions, we employed a suite of electrochemical techniques: potentiodynamic polarization (PDP), electrochemical impedance spectroscopy (EIS), and electrochemical frequency modulation spectroscopy (EFM). Furthermore, surface characterization of the metal after exposure to the inhibitor was conducted using ultraviolet-visible (UV-vis) spectroscopy and atomic force microscopy (AFM). To gain deeper insights into the AHMCC derivative's mode of action, adsorption mechanisms, and interactions at the metal-solution interface, we employed density functional theory (DFT) calculations to determine its electronic properties and reactivity descriptors. This combined approach of experimental data and theoretical modeling allowed for a more comprehensive understanding of the inhibitor's behavior.

## 2. Materials and methods

### 2.1. Solutions and materials

The carbon steel (C.STL) used in this study has the following chemical compositions (wt%): C 0.173, Mn 0.435, Cu 0.397, Ni 0.091, Cr 0.084, Si 0.0460, Co 0.040 and Fe the balance. The samples were cylindrical in shape welded with Cu-wire and embedded in resin with a (0.5 cm^2^) as the exposed surface area used for electrochemical experiments. Before use, the sample were abraded with various grades (800, 1200, 1500, and 2000) of emery papers. Then after, they were degreased in acetone, rinsed with ethanol, distilled water, dried with soft paper, and kept in a desiccator for use. HCl solution (0.5 mol L^−1^) was papered by diluting of (37% HCl) with deionized H_2_O. The different concentrations (15 × 10^−6^–30 × 10^−3^ mM) of (AHMCC) was papered in a (0.5 mol L^−1^) HCl solution.

### 2.2. Synthesis of (AHMCC) inhibitor

The FT-IR spectral analysis was employed using a Bruker FT-IR spectrometer (Invenio S, Germany). The spectral resolution was 4 cm^−1^, scans number was 64 and the wavenumber range of 400–4000 cm^−1^ was applied to the collection of IR spectra.^29^ FT-IR (*ν*, cm^−1^): 3314, 3051, 2929, 2215, 1583, 1566, 1506, 1311, 1291, 1216, 1161, 919. The ^1^H-NMR (DMSO-*d*_6_) spectrum was run on a Bruker (400 MHz) using TMS as an internal standard. ^1^H-NMR (DMSO-*d*_6_) (*δ*, ppm): 3.998 (s, 1H, CH), 6.956 (s, 2H, NH_2_), 6.977–8.229 (m, 10H, Ar–H), 8.730 (s, 1H, OH). Calcd for C_20_H_14_N_2_O_2_ (314.34): C, 76.42; H, 4.49; N, 8.91%. Found: C, 76.93; H, 4.62; N, 8.83%.

### 2.3. Antibacterial activity of (AHMCC)

#### 2.3.1. Agar well diffusion method

To evaluate the sample's antimicrobial activity, the agar well diffusion method was employed. This involved spreading a standardized amount of test bacteria across an agar plate.^30^ Then, a sterile cork borer or tip was used to aseptically punch a hole with a diameter of 9 mm in the agar. A set volume (100 μL) of the sample solution, prepared at the desired concentration (0.01 g dissolved in 1 ml DMSO), was then introduced into the well. After incubation under suitable conditions for the bacteria, the presence and diameter of a clear zone around the well (indicating inhibited microbial growth) were measured to assess the potency of the sample's antimicrobial effect.^31^

#### 2.3.2. Tested microbial strains

Gram-negative bacterial: *Escherichia coli* (ATCC 10536) and *Klebsiella pneumoniae* (ATCC 10031). Gram-positive bacteria: *Bacillus subtilis* (DMS 1088) and *Staphylococcus aureus* (ATCC 6538).

### 2.4. Electrochemical methods

The electrochemical cell used in this test consists of a platinum electrode as, an auxiliary electrode, C.STL as, a working electrode, and all potentials were recorded *versus* saturated calomel electrode (SCE) as a reference electrode. The working electrode was abraded with several of emery paper, rinsed with acetone and distilled water then it was dipped in the corrosive solution for 40 min until the open circuit potential (OCP) was obtained. The potentiodynamic polarization (PDP) was recorded in the potential range from −500 mV to +500 mV with 0.5 mV s^−1^ of the scan rate. The electrochemical impedance spectroscopy (EIS) test performed using alternating current (AC) signals of amplitude 10 mV peak to peak at (OCP) over the frequency range of 100 kHz–10 Hz. The polarization and impedance data were displayed in the form of Tafel and Nyquist plots, respectively, and the results are normalized to (0.5 cm^2^) surface area. The inhibition efficiency percentage (% IE_PDP_) was obtained using *I*_c_ values according to the equation as follows:^32^1
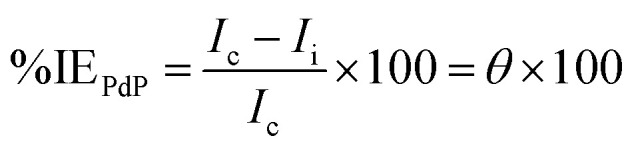
where, *I*_c_ and *I*_i_ refer to the corrosion current density (mA cm^−2^) without/with an inhibitor, respectively in test solutions. On the other hand, % IE_EIS_ was calculated on the basis of *R*_ct_ values using the following equation:^32^2
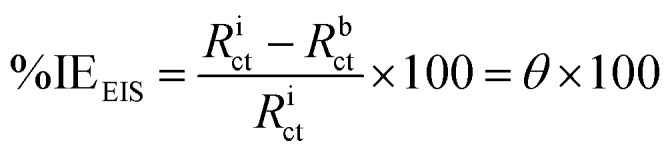
where, *R*^i^_ct_ and *R*^b^_ct_ are the charge transfer resistance values in (ohm cm^2^) with and without inhibitor in the test solutions, respectively. EFM measurements were conducted at two frequencies (2.0 and 5.0 Hz). The base frequency was 0.1 Hz and a potential disturbance signal of 10 mV. The (% IE_EFM_) was obtained using the previous eqn (1). The electrochemical tests were performed using a Gamry Potentiostat/Galvanostat ZRA analyzer (model PCI4G750).

### 2.5. UV-visible spectroscopy

#### 2.5.1. UV/visible spectra measurement

UV/visible spectral measurements were performed for (30 × 10^−3^ mM) of (AHMCC) solution only, 0.5 mol L^−1^ of HCl solution containing (30 × 10^−3^ mM) of (AHMCC) without/with dipping C.STL at 30 °C for 48 h using (PG instruments T80+ spectrometer).

### 2.6. AFM surface analysis

In preparation for the morphology study, the polished C.STL samples were immersed in test solutions at 30 °C for 48 h. The samples were then removed, rinsed with deionized water, and dried. Properties of the (AHMCC) protective layer on the C.STL surface were evaluated using AFM technique. After immersion in test solutions, the surface morphology of the C.STL samples were investigated through AFM (N9498S Agilent Technologies) analysis.

### 2.7. Theatrical calculations

The theoretical analysis of the investigated (AHMCC) was performed using the Gaussian program package (version 9.0; Pittsburgh, PA, USA). The geometry optimization, HOMO, LUMO configurations and related quantum chemical parameters obtained from analysis DFT level with B3LYP/6-311G ^++^ basis set in liquid phase.

## 3. Results and discussion

### 3.1. Synthesis of (AHMCC) inhibitor

**Scheme 1:**
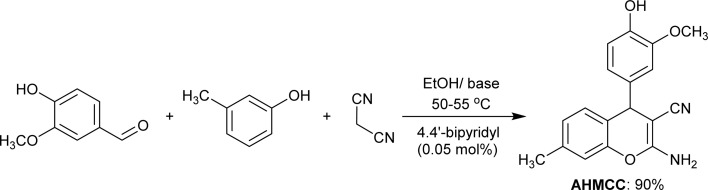
Synthesis of (AHMCC) inhibitor.

One-pot three-component reactions (3CRs) represent a concise and efficient strategy in organic synthesis, enabling the formation of complex molecules in a single reaction vessel from three diverse starting materials. This streamlined approach leverages a catalyst to orchestrate a cascade of reactions under optimized conditions, minimizing purification steps and potentially enhancing overall yield. The one-pot 3CR approach provides a promising method for synthesizing 2-amino-4*H*-chromene-3-carbonitrile.^33–35^ In this work, three-component one-pot reaction of vanillin with *m*-cresol and malononitrile gave the desired 2-amino-4*H*-chromene-3-carbonitrile (AHMCC) (Scheme 1). The reaction was accomplished in ethanol containing 4.4′-bipyridyl as a base under gently heating conditions (50–55 °C). Typically, moderate temperatures are employed to achieve efficient cyclization while minimizing side reactions. Ethanol is a polar protic solvent, meaning it has a hydroxyl group (OH) that can donate a hydrogen bond. This can be beneficial for solvating the starting materials, especially vanillin which has a hydroxyl group itself. However, the protic nature of ethanol might also lead to competing proton transfer steps that could complicate the desired cyclization. 4.4′-Bipyridyl is a neutral Lewis base. While it doesn't donate a proton like a Brønsted base, it can coordinate with Lewis acidic sites on the reactants, potentially activating them for further reaction. In the context of this pyran ring formation, 4.4′-bipyridyl might be complexing with the carbonyl group of vanillin, making it more susceptible to nucleophilic attack by the malononitrile. This streamlined approach offers efficiency and potentially reduces product loss compared to traditional multi-step syntheses.

#### 3.1.1. FT-IR spectral analysis

**Fig. 1:**
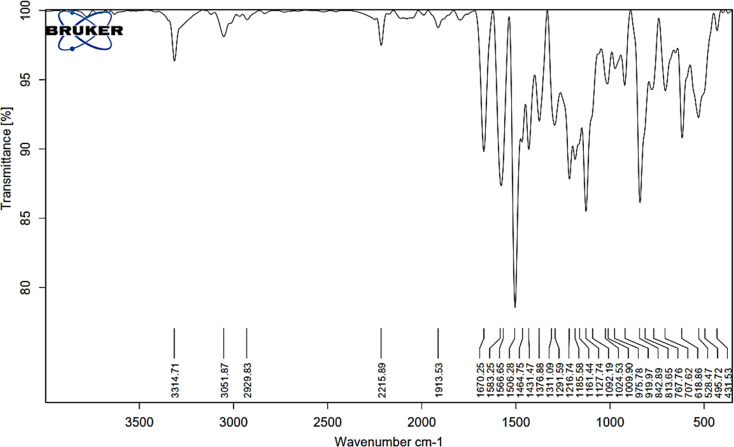
The FT-IR spectrum of AHMCC.

The FTIR spectral analysis results for AHMCC showed absorption bands at various wavenumbers, indicating the presence of different functional groups in the molecule (Fig. 1 and Table 1). Thus, the characteristic absorption bands at 3314, 3051, 2929 cm^−1^ are attributed to O–H (hydroxyl), C–H (aromatic and aliphatic) stretching vibrations. The absorption band at 2215 cm^−1^ is attributed to the presence of a nitrile (C≡N) functional group. The stretching vibrations of C=C group appeared at 1583 cm^−1^. In addition, the absorption bands due to aromatic ring vibrations appeared at 1566, and 1506 cm^−1^. The representative absorption bands at 1311, 1291, 1216, and 1161 cm^−1^ are due to C–N and C–O stretching vibrations. The presence of a broad peak around 3314 cm^−1^ suggests the presence of an O–H group, possibly from a hydroxyl group. The peaks at 3051 and 2929 cm^−1^ indicate C–H stretching, suggesting the presence of aromatic and aliphatic groups in the molecule. The sharp peak at 2215 cm^−1^ is a strong indicator of a nitrile (C≡N) functional group. The presence of multiple peaks between 1500 and 1600 cm^−1^ suggests the presence of an aromatic ring system. The peaks around 1311, 1291, and 1216 cm^−1^ could be due to C–N stretching vibrations in the amino group and C–O stretching vibrations. In particular, the FT-IR spectral analysis provides valuable information about the functional groups present in AHMCC.

**Table 1:** Experimental and theoretical FTIR spectral date of AHMCC

Experimental, *ν* (cm^−1^)	Theoretical, *ν* (cm^−1^)	Scale factor	Functional group	Interpretation
3314	3298	1.005	O–H (broad)	Hydroxyl group (possible)
3051	3048	1.001	C–H (aromatic, aliphatic)	Aromatic and aliphatic groups
2929	2915	1.005	C–H (aromatic, aliphatic)	Aromatic and aliphatic groups
2215	2204	1.005	C≡N	Nitrile group
1583	1590	0.996	Aromatic ring	Aromatic ring system
1566	1571	0.997	Aromatic ring	Aromatic ring system
1506	1498	1.005	Aromatic ring	Aromatic ring system
1311	1204	1.089	C–N/C–O	Amino group/C–O stretching
1291	1298	0.995	C–N/C–O	Amino group/C–O stretching
1216	1228	0.991	C–N/C–O	Amino group/C–O stretching
1161	1153	1.007	C–O	C–O stretching
919	930	0.988	Aromatic ring (out-of-plane)	Aromatic ring bending

**Fig. 2:**
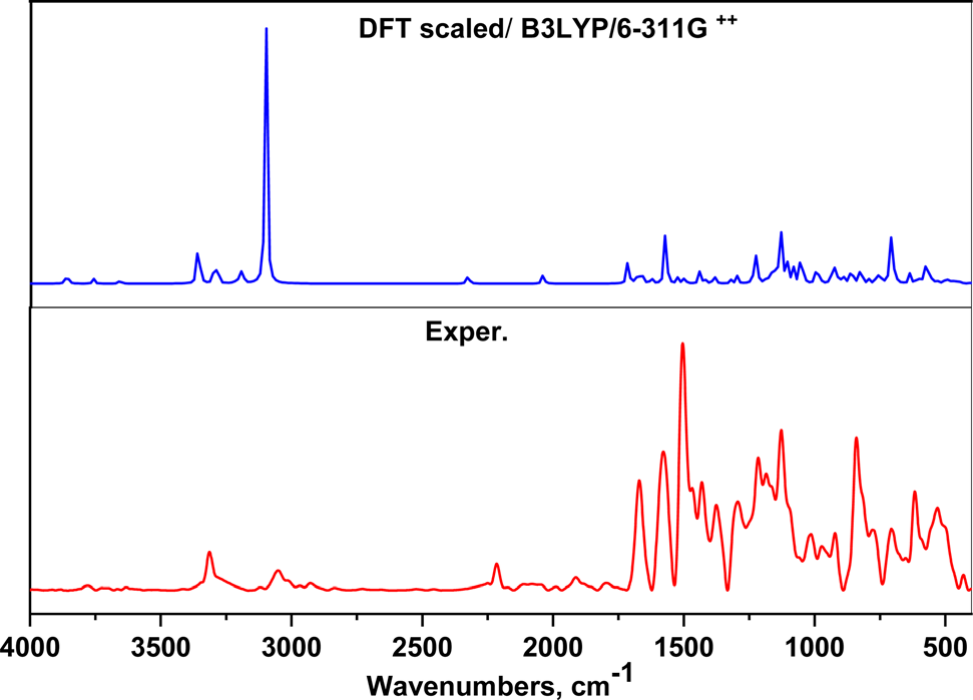
FTIR spectrum of AHMCC compared to the DFT scaled spectrum.

On the other hand, the vibrational frequencies (FTIR) for the optimized structure of AHMCC were determined and assigned by visual inspection using the Gauss View program. As depicted in Table 1, the frequency scaling factor is computed as the ratio of the experimental frequency to the theoretical frequency.^36^ Generally, there is good agreement between the experimental and theoretical FTIR frequencies, with most scaling factors being close to 1, indicating the accuracy of the theoretical model. Minor discrepancies arise due to computational limitations and non-linear vibration effects. The C–N/C–O stretching vibrations exhibit slightly larger deviations, indicating higher sensitivity to the computational method employed. Overall, the theoretical values align well with the experimental data, confirming the reliability of the calculations. The comparison between the experimental and calculated data shows good agreement. The differences might be because the calculations were for a free molecule in a vacuum, while the experiments were done on a solid sample (Fig. 2 and Table 1).

#### 3.1.2. The ^1^H-NMR spectral analysis

**Fig. 3:**
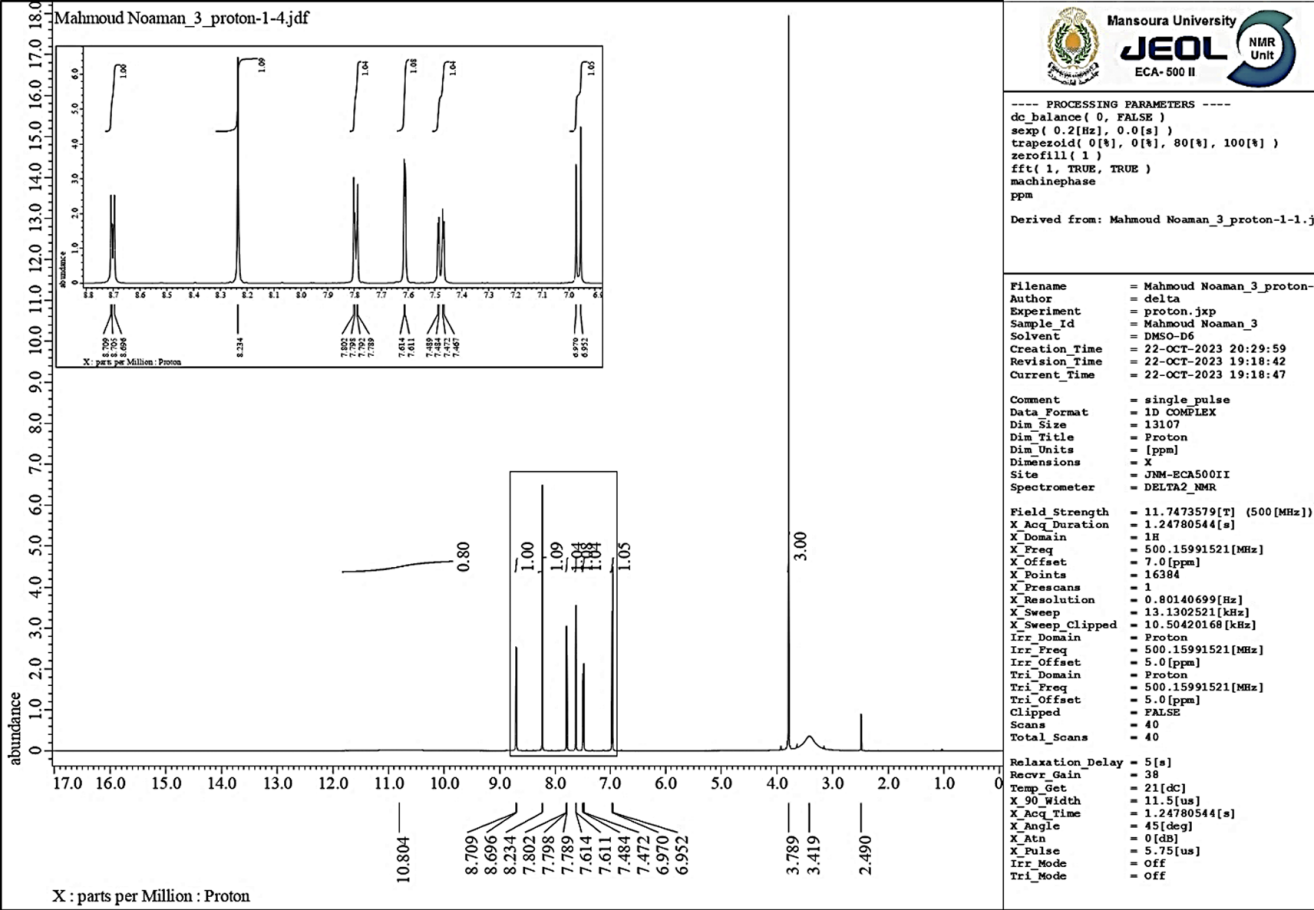
The ^1^H-NMR spectrum of AHMCC.

The ^1^H-NMR spectrum of AHMCC in DMSO (referenced to the solvent peak at ∼2.5 ppm) confirms the presence of the expected functional groups (Fig. 3). A broad singlet signal at 10.804 ppm integrates to 1H, attributed to the proton of the –OH group. A singlet peak at 7.15 ppm integrates to 2H, assigned to the 2-amino protons (NH_2_). A singlet peak at 3.8 ppm integrates to 3H, due to the protons on the C-3 methoxy group (OCH_3_). A singlet peak at 2.3 ppm integrates to 3H, assigned to the C-7 methyl protons (CH_3_). Multiplet peaks between 6.7 and 6.9 ppm represent the aromatic protons of the C-3 hydroxyphenyl group. A doublet peak at around 6.5 ppm (integration needed) is likely due to an aromatic proton on the C-4 methoxyphenyl group. The splitting patterns observed (singlets, multiplets) are consistent with the expected number of neighboring protons for each group. The chemical shifts of the protons also align with typical ranges for aromatic protons (6–8 ppm), amine protons (around 7 ppm), and methyl groups (around 2.3 and 3.8 ppm) next to electronegative oxygen. The absence of any significant peaks outside these expected regions suggests a relatively pure compound. Commonly, the ^1^H-NMR spectrum is consistent with the proposed structure of AHMCC (Table 2).

**Table 2:** The interpretation of the ^1^H-NMR spectrum in DMSO for AHMCC

Chemical shift (ppm)	Multiplicity	Interpretation
10.804	Broad singlet	Hydroxyl proton
7.15	Singlet	2-Amino protons
6.85	Singlet	Aromatic proton on the C-4 methoxy group
6.7–6.9	Multiplet	Aromatic protons on the C-3 hydroxyphenyl group
6.5	Doublet	Aromatic proton on the C-4 methyl group
3.8	Singlet	Protons on the C-3 methoxy group
2.3	Singlet	C-7 methyl protons

### 3.2. Antibacterial activity of (AHMCC)

**Fig. 4:**
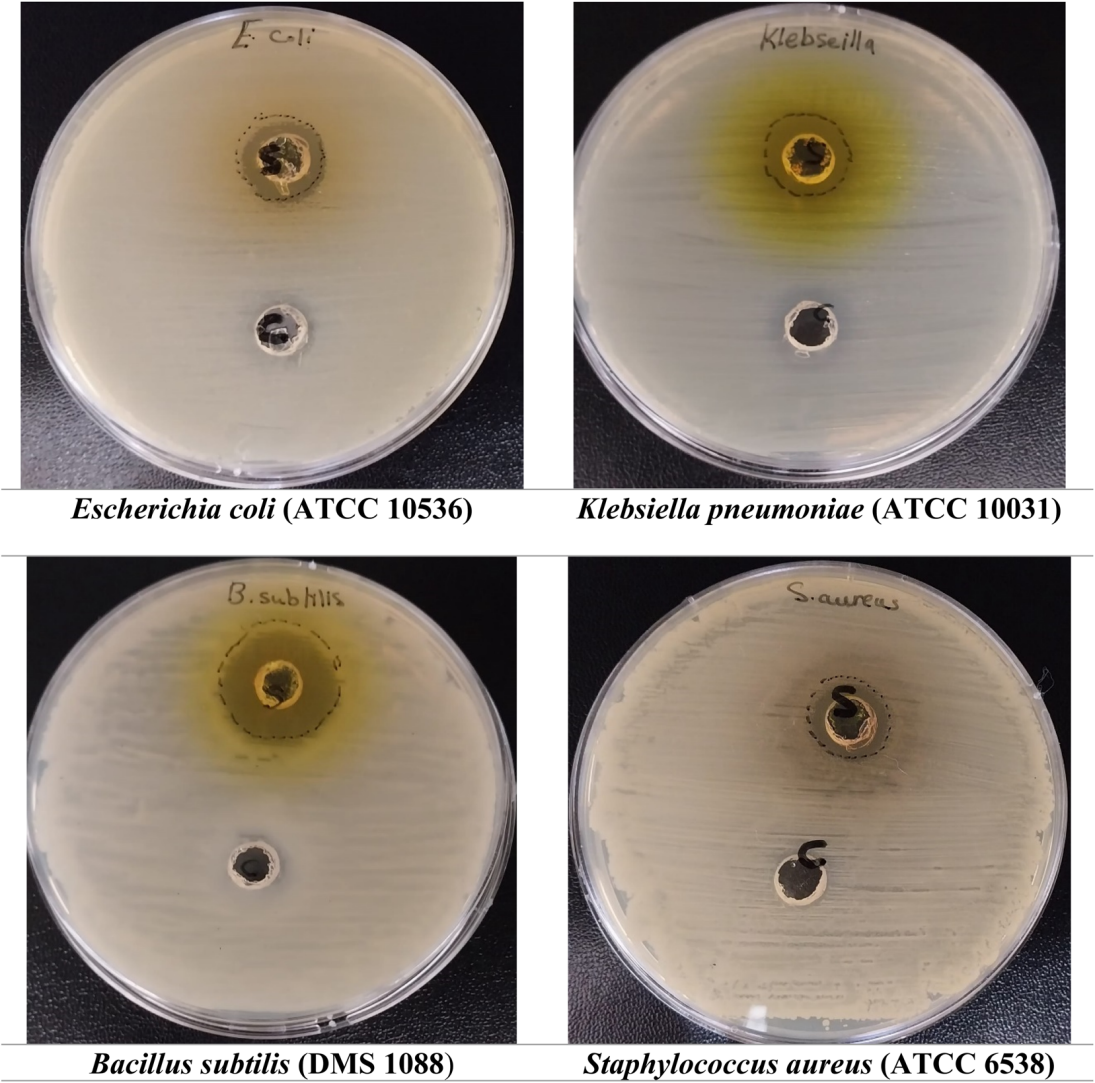
Clear zones around wells containing the tested compound indicate its inhibitory effect on bacterial growth.

The results in (Fig. 4 &Table 3) demonstrate the antibacterial activity of AHMCC against various bacterial strains. The test compound inhibited the growth of both Gram-negative (*E. coli* and *K. pneumoniae*) and Gram-positive bacteria (*B. subtilis* and *S. aureus*). Inhibition zone diameters ranged from 15.0 mm to 22.0 mm for the test compound, indicating moderate antibacterial potential. As expected, the control (DMSO) exhibited no inhibitory effect on any bacterial strains. In comparison to the test compound, Cefotaxime, the positive control, displayed a broader spectrum of activity with zone diameters ranging from 15.0 mm to 27.0 mm. These findings suggest that the test compound possesses promising antibacterial properties against a range of bacteria. While the activity was moderate compared to the broad-spectrum antibiotic Cefotaxime, it highlights the potential for further development of the test compound as an antimicrobial agent. In particular, the tested compound exhibited some variation in its inhibitory activity against the different bacterial strains tested (Table 3). While inhibition zone diameters ranged from 15.0 mm to 22.0 mm, the compound encouragingly inhibited both Gram-negative (*E. coli* and *K. pneumoniae*) and Gram-positive bacteria (*B. subtilis* and *S. aureus*). This demonstrates a broad spectrum of activity, with the highest effectiveness observed against *B. subtilis* (22.0 mm). These results, although showing some variation in potency, are promising as the compound managed to inhibit both major bacterial classifications, highlighting its potential for future development as a broad-spectrum antibiotic.

**Table 3:** Antibacterial activity (mm inhibition zone) of AHMCC compared to control (DMSO) and Cefotaxime (positive control) against Gram-negative and Gram-positive bacteria

Samples	Gram-negative bacteria		Gram-positive bacteria	
	*E. coli*	*K. pneumoniae*	*B. subtilis*	*S. aureus*
AHMCC	16.0 ± 0.36	15.5 ± 1.10	22.0 ± 1.49	15.0 ± 0.74
Control (DEMSO)	−ve	−ve	−ve	−ve
Cefotaxime (positive control)	23.0 ± 1.08	25.0 ± 1.05	27.0 ± 2.51	15.0 ± 0.92

The molecule's aromatic rings and lipophilic character could allow it to interact with and potentially disrupt the bacterial cell membrane.^37^ This disruption could lead to leakage of essential cellular components and ultimately cell death. The functional groups on the molecule, particularly the amino and nitrile groups, might be capable of binding to and inhibiting essential bacterial enzymes. This could disrupt metabolic pathways and hinder bacterial growth.^38^ The molecule's structure might allow it to interact with bacterial DNA or DNA replication processes. This could interfere with bacterial replication and prevent the growth of new cells. Structure–activity relationship (SAR) analysis involve investigating how modifications to the molecule's structure affect its potency and spectrum of action, potentially leading to optimized derivatives with enhanced antibacterial activity.^39–41^

The structure of AHMCC presents several interesting functionalities that could potentially influence its interaction with bacteria. The nitrile group (CN) draws electrons away from the rest of the molecule, potentially affecting its ability to form hydrogen bonds with other molecules. This could be important for how the molecule interacts with bacterial components. Additionally, the presence of a hydroxy group (OH) allows the molecule to act as a hydrogen bond donor, potentially enabling it to bind to specific sites on bacteria. In contrast, the methoxy group (OCH_3_) donates electrons, which may influence the overall polarity of the molecule and how it interacts with other molecules in its environment. Finally, the core structure, the 4*H*-chromene moiety, might be linked to some form of biological activity, but further research is needed to understand its specific role, if any, in inhibiting bacterial growth.

### 3.3. Electrochemical studies

#### 3.3.1. PDP tests

**Fig. 5:**
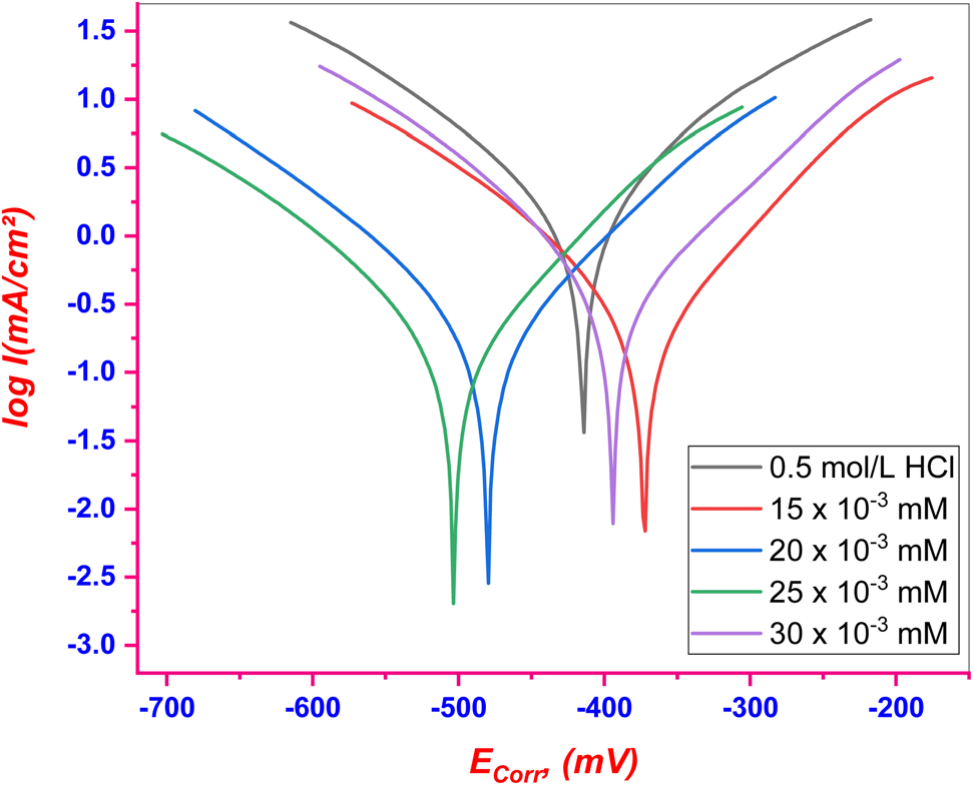
PDP curves for C.STL in test solution with/without of (AHMCC).

The PDP curves for C.STL in (0.5 mol per L HCl) solution with/without various concentrations of the (AHMCC) are illustrated in Fig. 5. The values of corrosion parameters, in addition, % IE_PDP_ and *θ* were calculated according to eqn (1), and are listed in Table 4. It is observed that from Fig. 4, and Table 4, the addition of (AHMCC) molecules to the corrosive media (0.5 mol per L HCl) shifted both anodic and cathodic branches of the PDP curves of HCS towards lower (*I*_c_) compared with the blank solution (0.5 mol per L HCl) at all investigated concentrations revealing retardation of both anodic and cathodic reactions and therefore, reduce the corrosion rate of the HCS the acidic medium, indicating successful % IE_PDP_.^42^ Inhibitors can be classified as anodic or cathodic based on an 85 mV change in (*E*_corr_) with respect to the blank solution (0.5 mol per L HCl).^43^ The (AHMCC) did not display such behavior, thus it functions by inhibiting both hydrogen gas evolution (cathodic reaction) and metal dissolution (anodic reaction). The (AHMCC) therefore seems to be a mixed-type inhibitor, inhibiting both the anodic and cathodic reaction, although it predominantly acts as an anodic inhibitor, as (*E*_corr_) shifted slightly towards anodic potential and anodic Tafel plot slope (*β*_a_) apparently decreased more than that recorded for the blank solution. The observed higher % IE_PDP_ at higher inhibitor concentrations can be attributed to adsorption of inhibitor molecules to form a stable protective film onto te active centers of C.STL.

**Table 4:** PDP kinetic corrosion parameters for (AHMCC)

Conc., (mM)	*I*_corr_, (mA cm^−2^)	−*E*_corr_, (mV)	*β*_a_, (mV dec^−1^)	−*β*_c_, (mV dec^−1^)	*θ*	% IE_PDP_
0	1.234	445	122.5	137.2	—	—
15 × 10^−3^	0.241	435	116.7	140.1	0.805	80.5
20 × 10^−3^	0.181	437	121.8	135.8	0.854	85.4
25 × 10^−3^	0.127	430	120.3	142.7	0.897	89.7
30 × 10^−3^	0.084	425	156.5	164.6	0.932	93.2

#### 3.3.2. EIS measurements

**Fig. 6:**
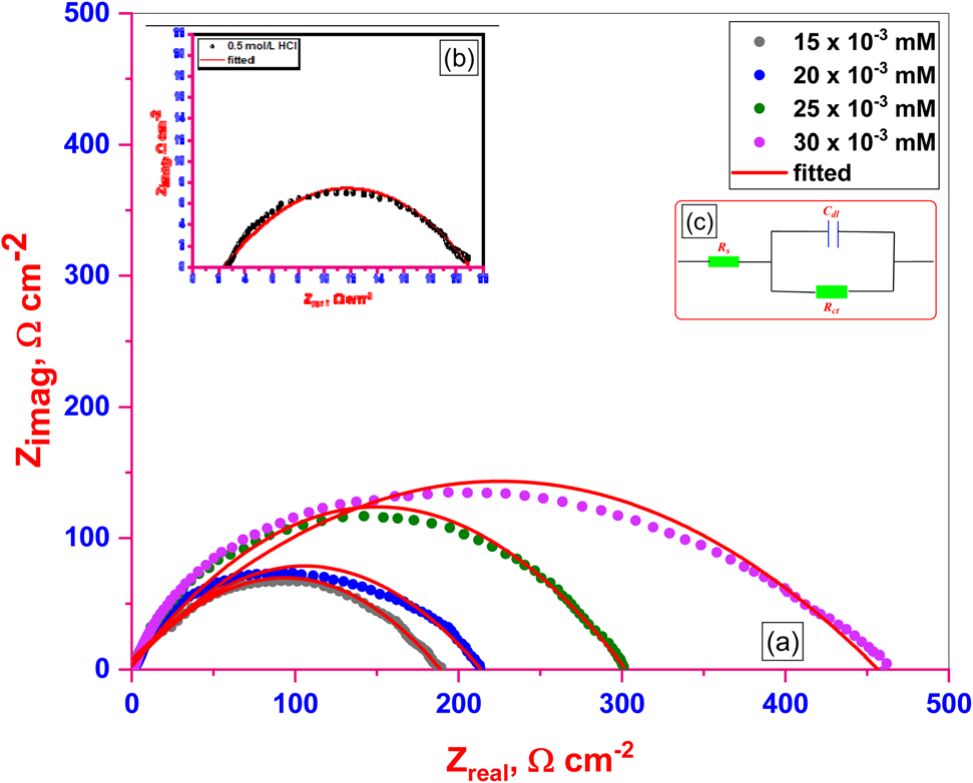
Nyquist plots (a) for C.STL in 0.5 M HCl solution with different concentrations of (AHMCC), (b) for C.STL in 0.5 M HCl solution respectively, using a suitable equivalent circuit model (c).

The behavior of C.STL corrosion in (0.5 mol per L HCl) solution was investigated in the absence and presence of different concentrations (AHMCC) molecules at 25 °C. Nyquist plots obtained from EIS measurements and the suitable equivalent circuit are illustrated in (Fig. 6a, and Fig. 6b), respectively. The charge transfer resistance (*R*_ct_) values obtained from fitted Nyquist plots, % IE_EIS_ values which calculated from the values of *R*_ct_ (using eqn (2)) and *θ* values are given in Table 5. It is clear that from Nyquist plots that spectra consist of one capacitive semicircle indicting that adsorption of inhibitors occurs by simple surface coverage and the (AHMCC) act as primary interface inhibitors, and the corrosion of C.STL is mainly controlled by charge transfer process.^44^ The capacitive semicircle size of C.STL increased significantly after addition of the (AHMCC) concentrations as a compared with the blank solution (0.5 mol per L HCl), indicating a decrease in the corrosion rate of C.STL and increasing the % IE_EIS_.^45^ In addition, it can be observed that the shape of the curves is similar in the absence or presence of different concentrations of (AHMCC), indicating that no alter in the corrosion mechanism. The values of (*R*_ct_) are listed in Table 5, showed that the addition of the (AHMCC) molecules in the corrosive media leads to increasing the value of (*R*_ct_) as a compered to the blank solution. This indicates that the (AHMCC) act as inhibitors through adsorption at the C.STL/solution interface which decreases their electrical capacities as they displace H_2_O molecules and other ions originally adsorbed onto the surface.^46^ However, increasing (*R*_ct_) value with inhibitor concentrations indicates that the amount of the inhibitor molecules adsorbed on the steel surface increases which form protective films on the metal surface resulting in increase in % IE_EIS_.^47^ On the contrary, as it can be seen from Table 5, the *C*_dl_ values tend to decrease with the increase of the concentration of the (AHMCC) in (0.5 mol per L HCl) solution. The decrease in the *C*_dl_, which can result from a decrease in local dielectric constant and/or an increase in the thickness of the electrical double layer, suggests that surfactants molecules function by adsorption at the metal/solution interface.^47^ Finally, the data of the % IE_EIS_ of the inhibitor get from EIS measurement were found to be an agreement with that calculated from measurement.

**Table 5:** EIS kinetic parameters for (AHMCC)

Conc., (mM)	*R*_s,_ (Ω cm^2^)	*R*_ct_, (Ω cm^2^)	*C*_dl_, (μF cm^−2^)	*θ*	% IE_EIS_
0	2.451	27.69	606.7	—	—
15 × 10^−3^	2.166	122.2	307.8	0.774	77.4
20 × 10^−3^	3.278	157.5	251.1	0.824	82.4
25 × 10^−3^	1.212	227.8	172.1	0.878	87.8
30 × 10^−3^	1.647	381.4	82.3	0.927	92.7

#### 3.3.3. EFM measurements

**Fig. 7:**
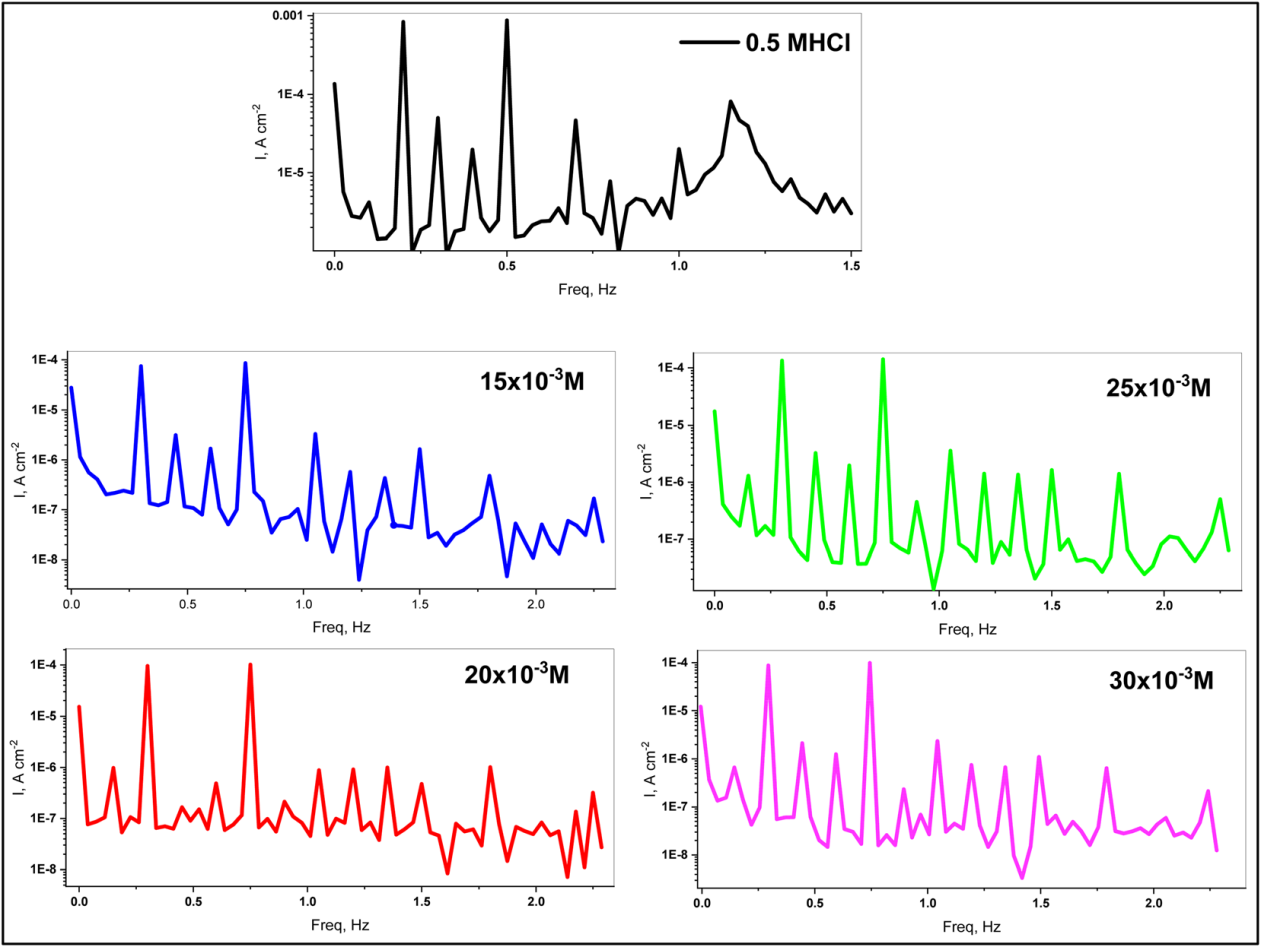
EFM spectra of C.STL electrode in 0.5 mol per L HCl solutions.

Electrochemical frequency modulation (EFM) is a non-destructive technique that provides direct corrosion current values without the need for prior knowledge of Tafel constants. It also has great strength due to the causality factor, which serves as an internal check on the accuracy of the EFM measurements. Fig. 7 represents the EFM spectra of C.STL electrodes in 0.5 M HCl solutions without (blank)/with various concentrations of (AHMCC) at room temperature. The EFM parameters as, *i*_corr_, *β*_a_, *β*_c_, and causality factors (CF-2) & (CF-3) are obtained from analysis of the larger peak of the EFM spectra (Fig. 7). In addition, (% I_EFM_), and (*θ*) values were calculated from eqn (1). As shown in Table 6, increasing the inhibitor concentrations leads to a decrease in (*i*_corr_) values, causing an increase in (% *I*_EFM_), and (*θ*) values, indicating that a protective layer developed on the metal surface. If the causality factors deviate significantly from the theoretical values of (2.0 & 3.0), it is possible to conclude that the data were influenced by noise. However, the causality factors are between (2.0 and 3.0), indicating that there is a causal relationship between the perturbation and response signals. The CF^2^ and CF^3^ in (Table 6) values are close to theoretical values (2.0 and 3.0, respectively), indicating that the experimental EFM results are valid.^48^ Furthermore, the EFM results are compatible with the PDP and EIS measurement results.

**Table 6:** EFM electrochemical corrosion parameters for C.STL

Conc., mol L^−1^	*i*_corr_, μA cm^−2^	*β*_a_, mV dec^−1^	*β*_c_, mV dec^−1^	CF^2^	CF^3^	*θ*	% *I*_EFM_
0.00	1.425	118.4	132.1	1.92	2.97	—	—
15 × 10^−3^	0.269	110.2	135.3	2.22	2.95	0.811	81.1
20 × 10^−3^	0.199	125.2	143.5	1.95	2.96	0.862	86.2
25 × 10^−3^	0.131	124.8	137.6	1.90	2.93	0.908	90.8
30 × 10^−3^	0.069	134.2	144.5	2.02	3.21	0.951	95.1

### 3.4. Adsorption isotherm

The chemical structure of the inhibitor, the charge on the metal surface, and the type of the corrosive medium all have an influence on the adsorption processes. Generally, physical (physisorption) and chemical (chemisorption) modes of adsorption can be taken into consideration.^49^ Experiments were performed to fit experimental data with different adsorption isotherms, including Temkin, Langmuir, Freundlich, Frumkin, Bockris–Swinkels, and Flory–Huggins, with Langmuir being the isotherm that is most usually employed in order to gain knowledge more about the adsorption process.

**Fig. 8:**
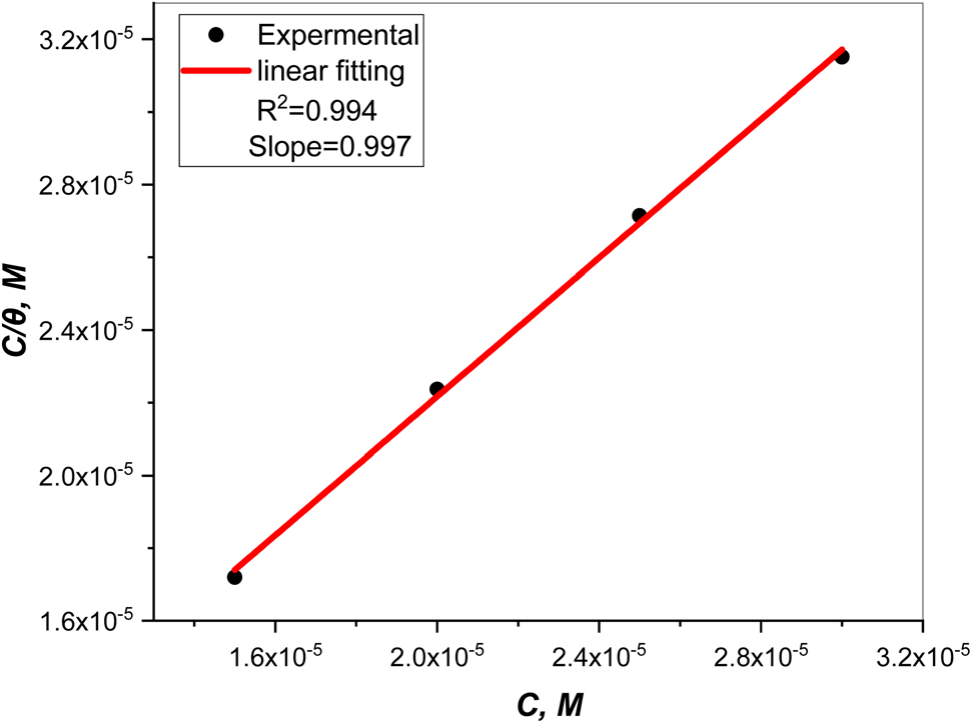
Plot of inhibitor concentrations (*C*_inh_) against (*C*_inh_/*θ*).

Plots of inhibitor concentrations (*C*_inh_) against (*C*_inh_/*θ*) at all the studied temperatures were done. As shown in Fig. 8, straight lines with approximately unit slopes and correlation coefficients (*R*^2^) of (0.997, and 0.994), respectively, were obtained.

This shows that the studied inhibitor's adsorption on the C.STL surface in the HCl solution (0.5 mol L^−1^) conforms to the Langmuir adsorption isotherm, which is described by the following equation:^50^3
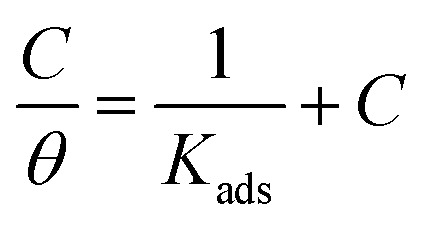
where and *K*_ads_ is the adsorptive equilibrium constant, and related to the standard free energy of adsorption 
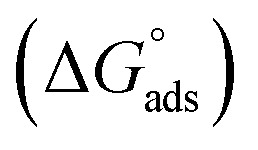
 acceding to the relation:^50^4
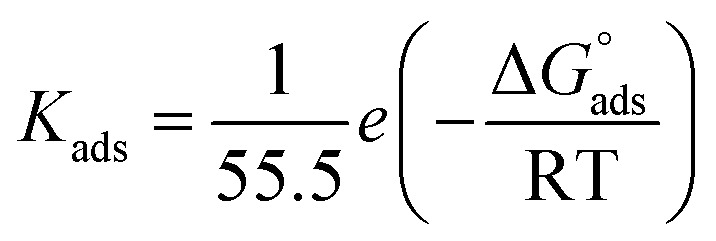
where, 55.5 values are water concentration (mol L^−1^), *K*_ads_ is the adsorptive equilibrium constant.

The *K*_ads_ value obtained from the intercept of the linear (Fig. 8) is high value (3.24 × 10^5^ mol L^−1^), indicating the strong adsorption of (AHMCC) molecules on the (C.STL) surface.^50^ The negative value of 
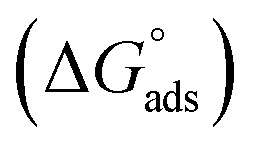
 obtained from eqn (4), suggest spontaneity of the adsorption process and stability of the adsorbed layer on the C.STL surface.^50^ Moreover, the value of 
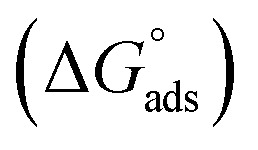
 obtained for (AHMCC) inhibitor in the current work is (−41.61 kJ mol^−1^), revealing the adsorption mechanism of the (AHMCC) is chemisorbed on C.STL surface in test solution.^50^

### 3.5. UV/visible spectra measurement

**Fig. 9:**
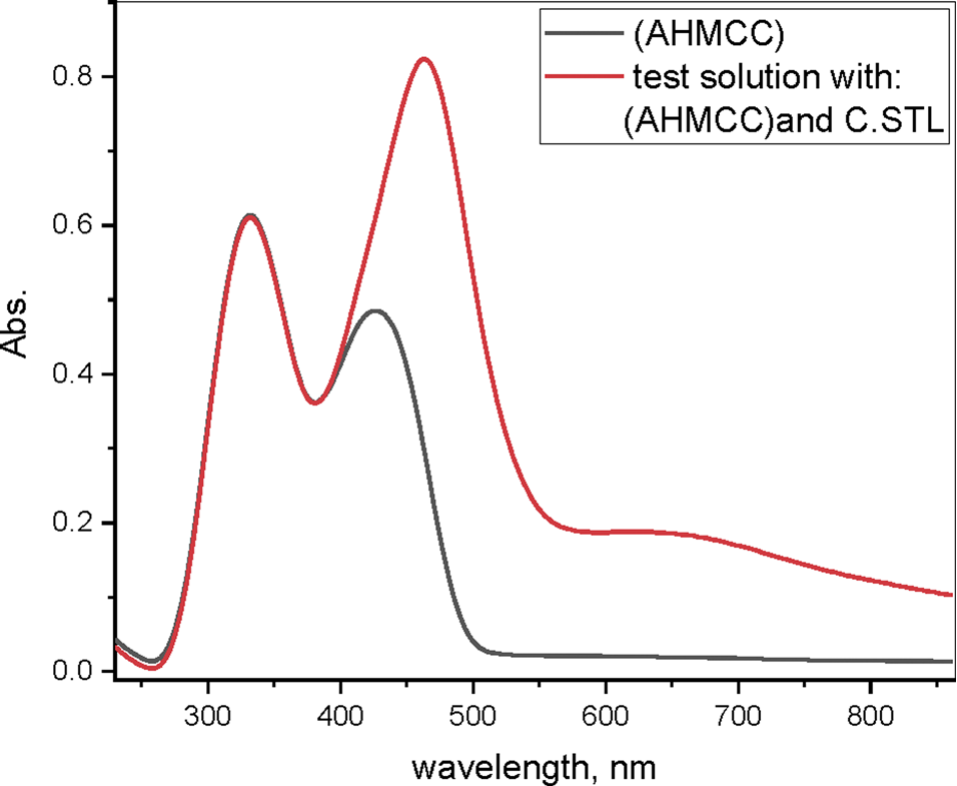
UV/visible spectra for the AHMCC solution (black color) and 0.5 M HCl solution containing AHMCC after C.STL immersion (red color) for 48 h at 30 °C.

The electron spectra of (AHMCC) solution (Fig. 9-black color), show peaks at *λ*_max_ (332, and 426 nm), respectively, suggesting the (π–π*), and (n–π*) transitions of the aromatic rings of inhibitor, respectively. But, the spectra of corrosive solution in the presence of (30 × 10^−3^ mM) of (AHMCC) after C.STL immersion in aggressive medium for 48 h are shown in (Fig. 9-red color) show new bands at *λ*_max_ (464 and 644 nm), respectively. This indicates the possibility of a complex formation between Fe^2+^ and (AHMCC) in (0.5 mol per L HCl) solution.^51^ In addition to, in our study, the 
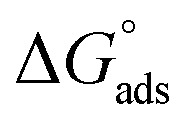
 value is (−41.61 kJ mol^−1^), confirming the chemical interaction of metal ion dissolution in an acidic solution with (AHMCC).

### 3.6. C.STL surface analysis (AFM)

**Fig. 10:**
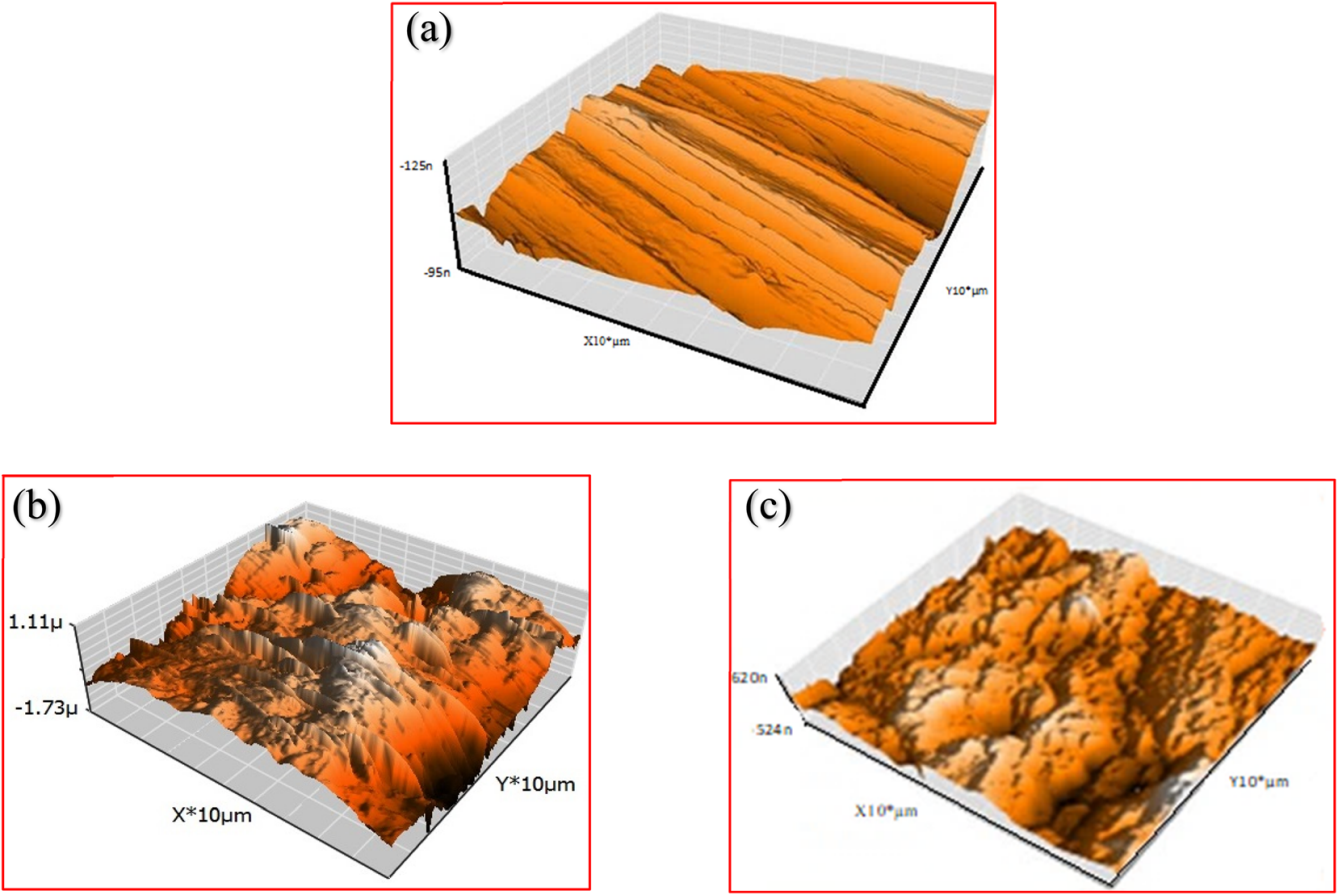
AFM images of free C.STL surface (a), in 0.5 mol per L HCl (b) and in 0.5 mol per L HCl with (AHMCC) (c).

Important information about the properties, morphology, roughness, of metal surfaces is provided by AFM.^52^Fig. 10 shows the three-dimensional (3D) AFM images of free C.STL surface sample (a), and was immersed for 48 hours in (0.5 mol per L HCl) without (b) and with (30 × 10^−3^ mM) of (AHMCC) solution (c). The roughness average (*R*_a_) and root mean square roughness (*S*_q_) were obtained from AFM spectroscopy analysis.

Fig. 10 shows that the free C.STL surface is smooth and homogeneous (Fig. 10a), and the corresponding (*R*_a_) and (*S*_q_) is (65.2 and 102 nm), respectively. While, the C.STL surface sample was immersed in (0.5 mol per L HCl) solution (Fig. 10b) shows a wide cavity was formed on the metal surface and the corresponding (*R*_a_) and (*S*_q_) are a large value (610.2 and 750.1 nm), respectively. On the hand, upon addition of (30 × 10^−3^ mM) of (AHMCC) (Fig. 10c), the surface significantly improved attributed to formation of more compact protective film,^53^ and the roughness (*R*_a_) and (*S*_q_) are of C.STL decreased to (95.4 and 117.5 nm), respectively. These visual data obtained from AFM analysis support the results get from the electrochemical measurements.

### 3.7. Theoretical analysis

**Fig. 11:**
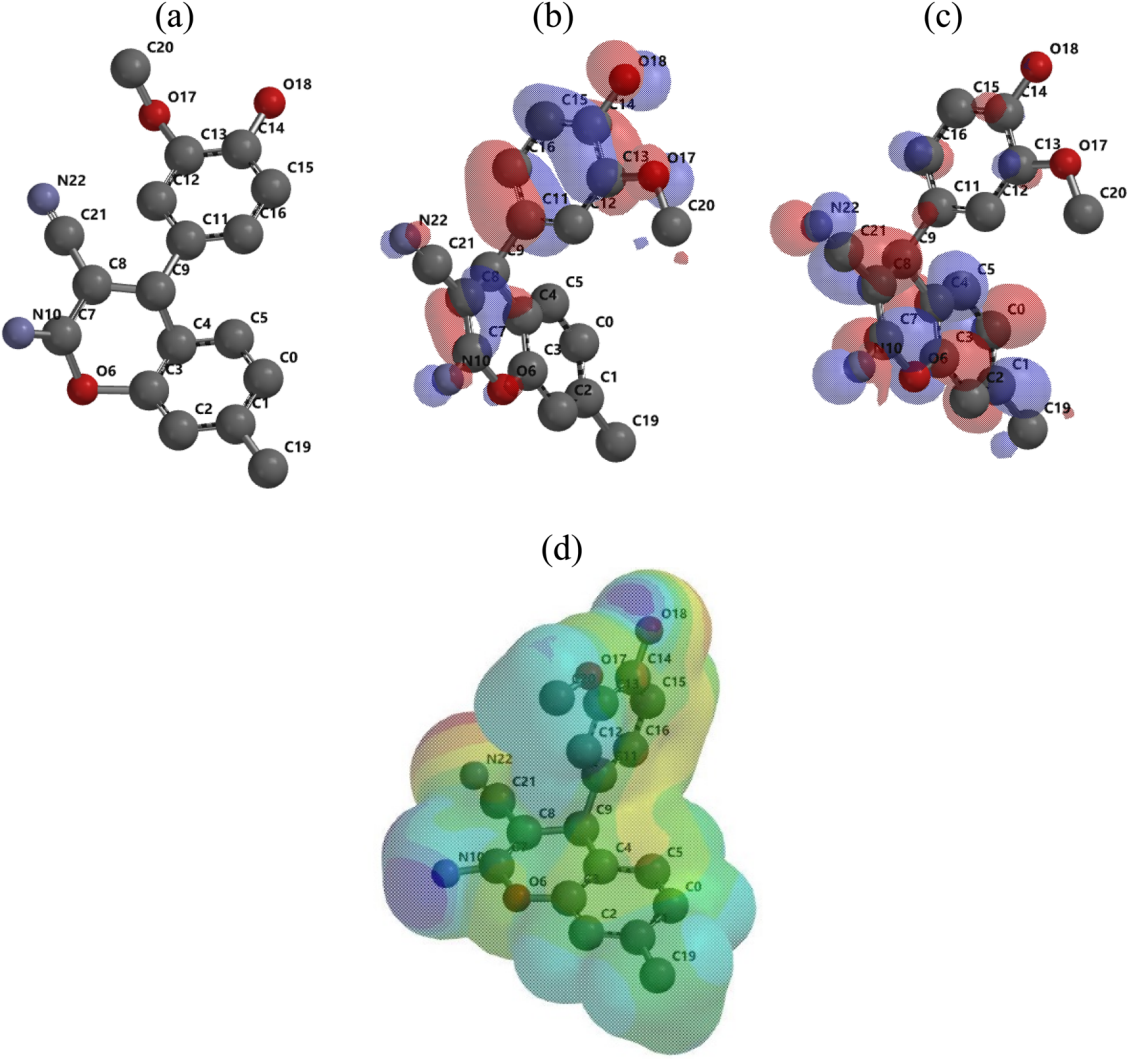
Optimized geometrical structure (a); HOMO (b), LUMO (c) and ESP (d) for (AHMCC).

The optimized geometry, HOMO, and LUMO orbitals and ESP of the investigated AHMCC inhibitor were obtained using DFT method, and shown in Fig. 11. The distribution HOMO of AHMCC molecule is concentrated on the phenyl, –OCH_3_, and –C=O groups, and is scattered on pyran ring. On the other hand, it is observed that the distribution of LUMO is located on phenyl ring fused with pyran ring, –CN, and –NH_2_ is scattered on the pyran ring. The quantum chemical parameters of (AHMCC) molecule were derived from DFT analysis are listed in Table 7. The high value of *E*_HOMO_ (−5.532 eV) representing its extraordinary ability to deliver the electrons to the C.STL surface.^54^ On the contrary, the lowest value of *E*_LUMO_ (−0.583 eV) shows the highest capacity to donate electrons to C.STL. On the other hand, the low-level value of Δ*E*_gap_ (2.569 eV) represents its strong power of the [C.STL–AHMCC] complex. The (Δ*N*) for AHMCC is a negative value (−0.206), clarifying the electron transfer from copper metal to AHMCC molecule (*i.e.*, AHMCC molecule is electrons acceptor).^55^ The red area on the ESP map in Fig. 11d represent the region which is related to the (nucleophilic attack). While, the green area to blue area responsible for the electrophilic attack. According to ESP analysis of (Fig. 11d), the nucleophilic attack is existing on NH_2_, pyran ring. While, the electrophilic attack is existing across the whole (AHMCC) molecule.

**Table 7:** Quantum chemical parameters of (AHMCC)

Energy	Symbol and equation	Value, eV
HOMO energy	*E*_HOMO_	−5.532
LUMO energy	*E*_LUMO_	−0.583
Energy gap	Δ*E*_gap_ = *E*_LUMO_ − *E*_HOMO_	4.949
Ionization potential	*I* = −*E*_HOMO_ (eV)	5.532
Electron affinity	*A* = −*E*_LUMO_ (eV)	0.583
Global hardness	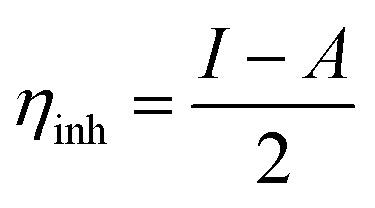	2.4745
Electronegativity	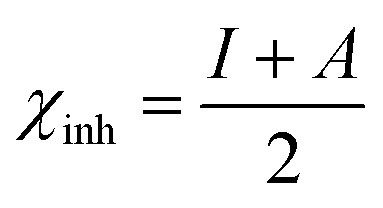	5.8235
The electrons transferred	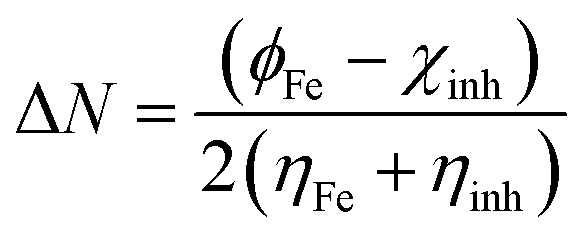 *ϕ*_Fe_ is the work function of Fe surface (4.8 eV)	−0.21

**Fig. 12:**
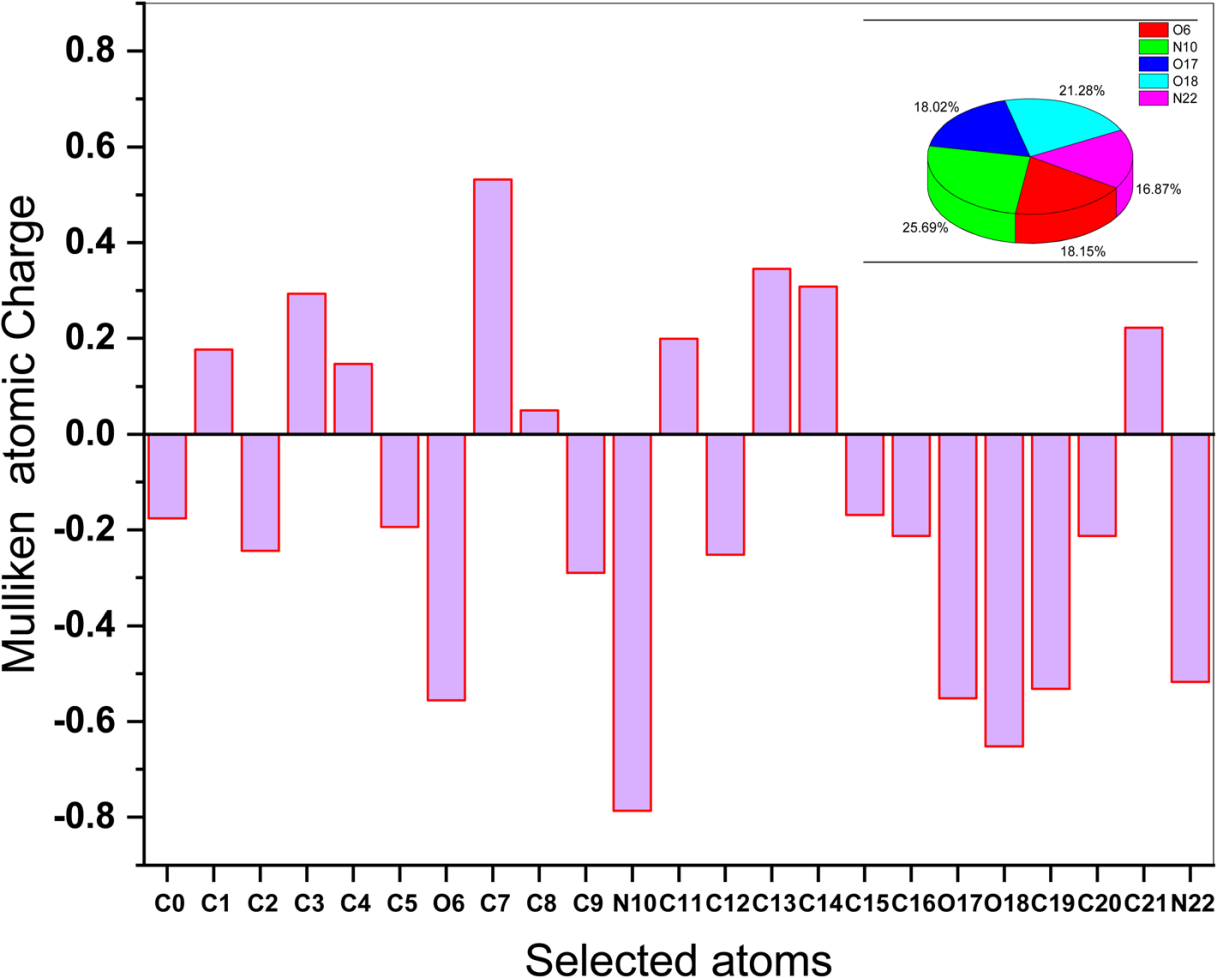
Mulliken atomic charge of (AHMCC).

The Mulliken atomic charges of the (AHMCC) molecule are represented in Fig. 12, showing the heteroatoms have negative charges which are responsible for electrons donation to the unoccupied d-orbitals of the C.STL metal.^56^ On the contrary, the heteroatoms have positive charges and can accept electrons from the 3d orbital of metal atoms. As shown from (Fig. 12), it is found that the (O6, N10, O17, O18 and N22) have a more negative charge, indicating that these atoms can take part in donor–acceptor interactions with surface metallic centers, thereby decreasing the corrosion rate on metal in the test solution.^57^

### 3.8. Corrosion inhibition mechanism & comparison with other studies

**Fig. 13:**
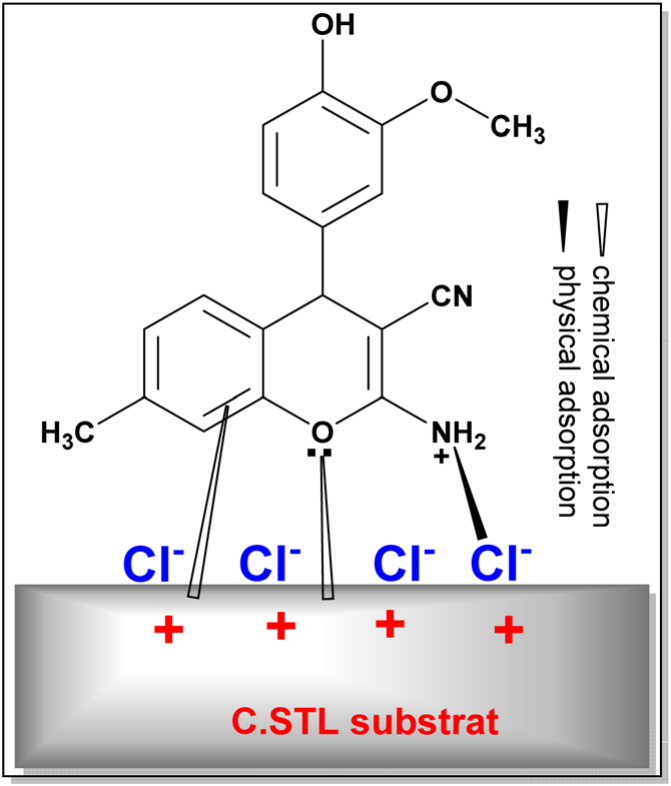
The adsorption mode of (AHMCC) on the C.STL surface in acidic solution.

EIS, PDP and AFM measurements revealed that the (AHMMC) inhibitor considerably retard/inhibit the metal corrosion in acidic conditions. The corrosion inhibition property of the (AHMMC) derivative can be attributed to the presence of heteroatoms and π-electrons on phenyl ring. These factors play the vital role in the adsorption of the inhibitor and the formation of co-ordinate bond with metal. The adsorption of inhibitor on the steel surface can occur either directly *via* the interactions between the electron pairs of heteroatoms and π-electrons of the aromatic rings in the (AHMMC) and the vacant d-orbitals of metal surface atoms (called as chemical adsorption). On the other hand, in the acidic medium the (AHMCC) molecules change into the protonated form, which promotes the electrostatic interaction with negatively charged metal produced *via* reabsorbed Cl^−^ ions (called as physical adsorption) (Fig. 13).^58^

This research examined the effectiveness of (AHMCC) as a corrosion inhibitor for carbon steel (C.STL) exposed to an acidic environment. To assess its performance relative to existing solutions, we compared (AHMCC) with inhibitors studied in previous research.^52–56^Table 8 summarizes this comparison, showcasing the percentage inhibition (% *I*) achieved by each inhibitor. This table provides valuable insights into how (AHMCC) stacks up against other corrosion inhibitors. It reveals that compound CN-1 reigns supreme with the highest % *I* (97.5%) when tested on N80 steel in a 15% hydrochloric acid (HCl) solution. This suggests exceptional potential for CN-1 in mitigating corrosion under these specific conditions. Other inhibitors, including CMCC, CN-2, and 2-mercaptothiazoline (2-MTZL), also demonstrate impressive performance, exceeding a 90% inhibition rate. Our own inhibitor, AHMCC, achieves a competitive % *I* of 91.0 when tested on carbon steel in a 0.5 M hydrochloric acid solution using PDP, EIS, and EFM methods. However, it's important to acknowledge that the table compares results obtained under various conditions, including the type of metal, electrolyte, and testing methods employed. This variation makes a direct side-by-side comparison less precise. Additionally, factors beyond % *I*, such as cost-effectiveness and environmental impact, are not included in the table. These factors can significantly influence the choice of inhibitor for a particular application. In conclusion, the table provides valuable insights into the performance of various corrosion inhibitors, including AHMCC. However, for the most informed decision, selecting the optimal inhibitor necessitates considering the specific use case, test conditions, and other relevant factors alongside the % *I* value.

**Table 8:** Comparison of (AHMCC) performance with previous studied corrosion inhibitors

Inhibitor	Metal/Electrolyte	Methods	% *I*	Ref.
(*E*)-2-Amino-4-((3-cyano-8-methoxy-2*H*-chromen-2-ylidene)amino)-8-methoxy-4*H*-chromene-3-carbonitrile (CMCC)	Mild steel/15% HCl	Wt. loss, PDP, EIS	93.6	59
(*E*)-2-Amino-4-((2-amino-3-cyano-8-methoxy-4*H*-chromen-4-yl)(cyano)methylene)-8-methoxy-4*H*-chromene-3-carbonitrile (CYCC)			91.8	
5-Amino-9-hydroxy-2-(4-vinylphenyl)-1,11b-dihydrochromeno[4,3,2-*de*][1,6]naphthyridine-4-carbonitrile (CN-1)	N80 steel/15% HCl	Wt. loss, PDP, EIS	97.5	60
5-Amino-9-hydroxy-2-(4-methoxyphenyl)chromeno[4,3,2-*de*][1,6]naphthyridine-4-carbonitrile (CN-2)			97.4	
5-Amino-9-hydroxy-2-phenylchromeno[4,3,2-*de*][1,6]naphthyridine-4-carbonitrile (CN-3)			95.4	
(*E*)-3-(((4-Acetamidophenyl)imino)methyl)-6-methyl-4*H*-chromen-4-one (AIMCH)	Carbon steel/15	Wt. loss, PDP, EIS	92.2	61
(*E*)-3-(((4-methoxyphenyl)imino)methyl)-6-methyl-4*H*-chromen-4-one (MIMCH)			85.4	
1,3-Dioctadecylimidazolium bromide (ImDC18Br)	Mild steel/1 M H_2_SO_4_	PDP	90.0	62
*N*-Octadecylpyridinium bromide (PyC18Br)			91.2	
2-Mercaptothiazoline (2-MTZL)	Low carbon steel/3 M H_3_PO_4_	Wt. loss	93.0	63
Cetyl pyridinium chloride (CPYCl)			88.0	
2-Amino-4-(4-hydroxy-3-methoxyphenyl)-7-methyl-4*H*-chromene-3-carbonitrile (AHMCC)	Carbon steel/0.5 M HCl	PDP, EIS, EFM	91.0	Our result

## 4. Conclusions

This study successfully synthesized and evaluated a novel compound, 2-amino-4-(4-hydroxy-3-methoxyphenyl)-7-methyl-4*H*-chromene-3-carbonitrile (AHMCC), for its dual functionality: antibacterial activity and corrosion inhibition of carbon steel (C.STL) in 0.5 M HCl solution. AHMCC demonstrated remarkable antibacterial efficacy and achieved an impressive 93.4% corrosion inhibition efficiency for C.STL. Electrochemical studies revealed that AHMCC acts as a mixed-type inhibitor, effectively suppressing corrosion through an adsorption mechanism. This finding was further supported by the close agreement between the experimental and theoretical CF_2_ and CF_3_ values obtained from Electrochemical Frequency Modulation (EFM) measurements. Langmuir isotherm analysis, based on Electrochemical Impedance Spectroscopy (EIS) data, corroborated the formation of an adsorbed inhibitor layer on the C.STL surface. Ultraviolet-visible spectroscopy and Atomic Force Microscopy (AFM) provided visual confirmation of this adsorption phenomenon. Furthermore, Density Functional Theory (DFT) calculations yielded quantum chemical parameters that aligned well with the experimental observations, strengthening the validity of the results. In conclusion, this study presents AHMCC as a promising candidate for combating both bacterial growth and metal corrosion in acidic environments. The combined approach of experimental evaluation and theoretical modeling provides a comprehensive understanding of AHMCC's inhibitory mechanisms, paving the way for its potential application in industrial settings.

## Data availability

The data that support the findings of this study are available from the corresponding author upon reasonable request.

## Conflicts of interest

The author declares no conflict of interest.
